# The cn/cn dwarf mouse. Histomorphometric, ultrastructural, and radiographic study in mutants corresponding to human acromesomelic dysplasia Maroteaux type (AMDM)

**DOI:** 10.1186/1471-2474-15-347

**Published:** 2014-10-15

**Authors:** Frederic Shapiro, Lauren Barone, Andrew Johnson, Evelyn Flynn

**Affiliations:** Department of Orthopaedic Surgery, Orthopaedic Research Laboratory, Boston Children’s Hospital, 300 Longwood Avenue, Boston, MA 02115 USA

## Abstract

**Background:**

The cn/cn dwarf mouse is caused by a loss-of-function mutation in the natriuretic peptide receptor 2 (NPR-2) gene which helps positively regulate endochondral longitudinal bone growth. The gene mutation corresponds to that in the human skeletal dysplasia Acromesomelic Dysplasia Maroteaux type (AMDM). This study assesses histomorphometric, ultrastructural and radiographic correlates of the growth abnormality.

**Methods:**

Ten litters of cn/cn and cn/+littermates at ages ranging from 2.5 to 6.5 weeks were studied by skeletal radiographs, histomorphometry and physeal ultrastructure. Skeletal radiographs were done on 2 cn/cn and 2 cn/+littermates at 5 weeks of age. Humeral, femoral, and tibial lengths were measured from 34 intact bones (17 cn/cn, 17 cn/+) at 2.5 to 6.5 weeks. Growth plate histomorphometry in 50 bones (26 cn/cn and 24 cn/+) determined the hypertrophic zone/entire physeal cartilage ratios in 204 sections (87 cn/+, 117 cn/cn) at 3 time periods (2.5-3, 4–4.5, and 6–6.5 weeks). Electron microscopy assessed 6 cn/cn and 6 cn/+age and site-matched physeal cartilage.

**Results:**

Cn/cn mice were two thirds the size of the cn/+. Cn/cn bones were normal in shape or only minimally deformed except for the radius with mid-diaphyseal bowing. Length ratios of cn/cn humeri, femurs, and tibias were a mean of 0.65 (±0.03, n = 34, 17 ratios) compared to cn/+bones. The main physeal abnormality was a markedly shortened hypertrophic zone with the ratio of hypertrophic zone to entire physis 0.17 (±0.063) in the cn/cn and 0.30 (±0.052) in the cn/+mice. Ratio assessments were similar comparing humeral, femoral, and tibial growth plates as were ratios from each of the 3 time periods. Ultrastructural assessments from the resting zone to the lower hypertrophic zone-metaphyseal junction showed no specific individual cell abnormalities in cn/cn compared to cn/+physes.

**Conclusions:**

The disorder causes a shortened physeal hypertrophic zone but normal ultrastructure of cn/cn chondrocytes points to abnormality primarily affecting the hypertrophic zone rather than a structural cell or matrix synthesis problem.

**Electronic supplementary material:**

The online version of this article (doi:10.1186/1471-2474-15-347) contains supplementary material, which is available to authorized users.

## Background

The cn/cn skeletal dysplasia mouse developed following a spontaneous mutation in strain AKR/J in the mouse colony at the Jackson Laboratory, Bar Harbor ME. Short stature, abnormal growth plate characteristics, and an autosomal recessive pattern were described in the initial report [1]. Slowed growth of the femur and tibia [2], abnormal histologic appearance of the growth plates [3–10], and extent of involvement of various regions of the physes [3, 7, 9, 10] were defined. Affected cn/cn mice were only 60-70% the size of unaffected littermates, the bones that developed via the endochondral sequence were 60%-70% the length of normals with intramembranous bone unaffected, and in the histologically abnormal growth plates the shortened hypertrophic chondrocyte zone was the most affected part.

The cn/cn mouse was originally named as an achondroplastic mouse but once human achondroplasia was found to be due to mutations in the gene encoding fibroblast growth factor receptor 3 [11, 12] the cn/cn mouse was no longer considered analogous to human achondroplasia and came to be described simply as cn/cn. Several years later the defect site in the cn/cn mouse was mapped to chromosome 4 [13, 14] and the abnormality was identified as a loss-of-function mutation in natriuretic peptide receptor 2 (Npr2) gene which encodes a receptor for the natriuretic peptide that positively regulates endochondral longitudinal bone growth [14]. Mutations in the transmembrane natriuretic peptide receptor NPR-B have also been defined in a human skeletal dysplasia, acromesomelic dysplasia Maroteaux type (AMDM) [15], whose locus had been mapped to chromosome 9 [16, 17]. Based on these corresponding mouse/human findings, assessment of the cn/cn mouse has been undertaken for a clearer understanding of how the gene mutation leads to structural physeal abnormality and growth deformity. We outline differences between the affected cn/cn mice and their non-affected cn/+littermates regarding: i) overall growth and length of individual bones to assess whether any skeletal regions are preferentially affected; ii) quantitative physeal findings using light microscopic histomorphometry; iii) shape of individual bones and skeletal regions as determined radiographically; and iv) ultrastructural assessments of physeal chondrocytes.

## Methods

**Figure 1:**
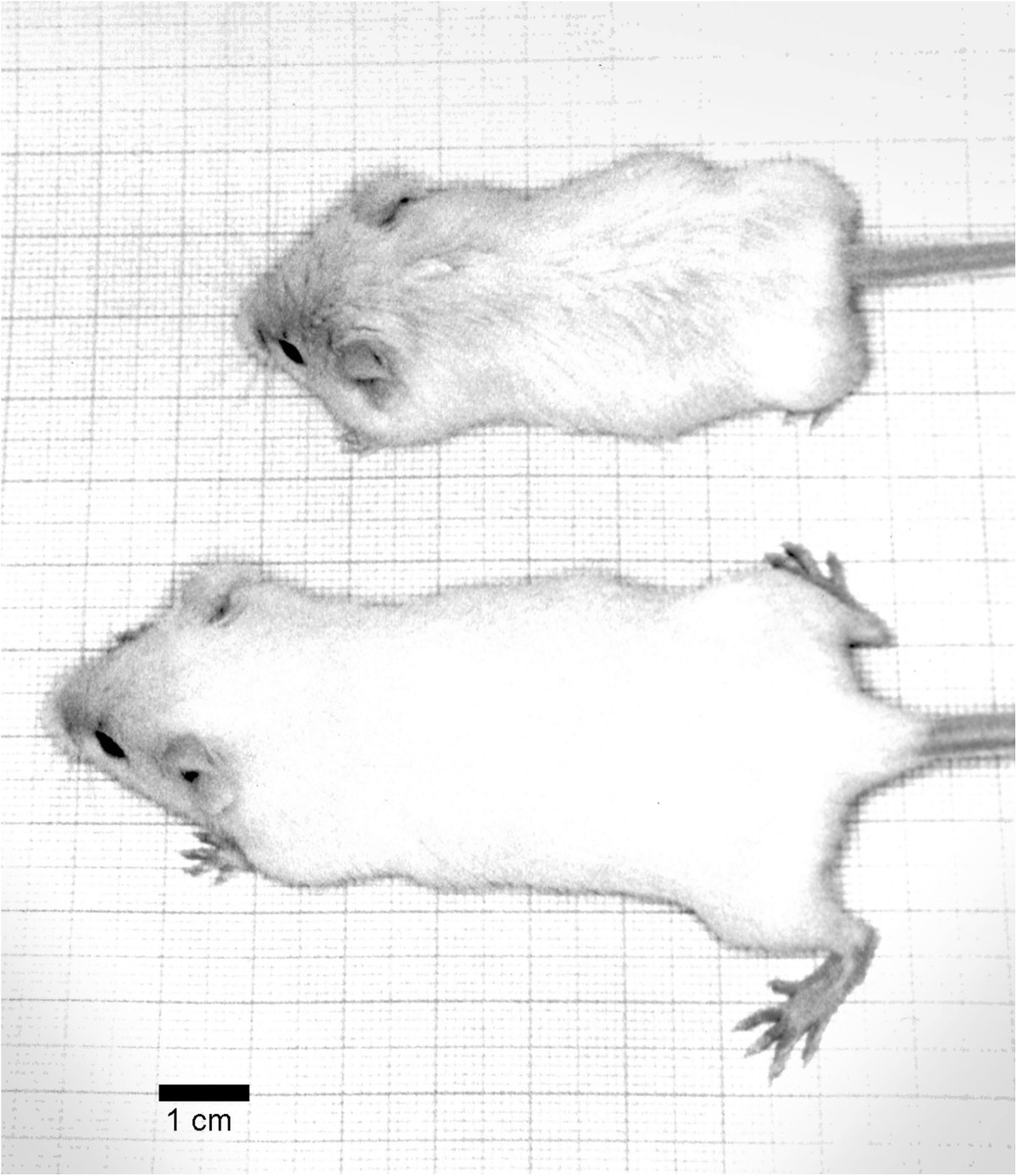
**Comparative Mouse Photographs.** Photograph illustrates cn/cn mouse (top) and cn/+ mouse (bottom) from the same litter. The crown-rump length of the smaller cn/cn mouse is 70% that of the normal cn/+.

The cn/cn mice were obtained from Jackson Laboratories, Bar Harbor, ME. Ten separate litters were studied at ages 2.5 weeks (3), 3 weeks (2), and one each at 4, 4.5, 5, 6, and 6.5 weeks. Recognition of affected (cn/cn) and non-affected (cn/+) littermates was evident at 2.5 weeks of age (Figure 1). All animal work was conducted according to relevant national and international guidelines. The research was approved by the Boston Children’s Hospital Animal Research Committee. The animals were sacrificed by intraperitoneal injection of sodium pentothal.

### Radiographic assessments

Standardized anteroposterior radiographs of the entire skeleton were made on 2 affected cn/cn and 2 non-affected cn/+littermates at 5 weeks of age using Faxitron (General Electric) at 32 kilovolts with exposure time 48 seconds. Radiographs were taken following removal of skin, heart, lungs, abdominal viscera, sternum and anterior rib cage. The four littermates, two cn/cn and two cn/+, were radiographed on the same exposure for accurate comparison. Additional anteroposterior spinal radiographs were made from cn/cn and cn/+littermates at 2.5 weeks of age. Comparative assessments in cn/cn and cn/+littermates were made of: i) radiologic appearance of the bones, ii) number of ribs and vertebrae including the tail vertebrae, and iii) (where uniform positioning allowed) relative percentage differences of lengths of radius, ulna, metacarpal and metatarsal and corresponding phalangeal bones, and pelvis (iliac crest to distal tip of ischium) and interpedicular widths of the L1 and L6 vertebrae.

### Humeral, femoral and tibial lengths

Humeri, femurs and tibias were removed and measurements of entire intact bones were performed using a millimeter ruler and an 8 × loupe. Humeri were measured from the proximal humeral head articular surface to the distal condylar articular surface, femurs from the proximal femoral head articular surface to the lateral condyle articular surface, and tibias from the proximal articular surface to the distal articular surface. Affected (cn/cn) and non-affected (cn/+) littermates served as age matched comparisons to establish ratios of mutant to normal lengths. Assessments were made at 2.5, 3, 4, 4.5, 6, and 6.5 weeks of age in 34 bones from age-matched littermates.

### Weights

Cn/cn and cn/+littermates were weighted at 4 weeks of age (3 from each group) and 6 weeks of age (4 from each group).

### Light microscopy

Entire long bones or their epiphyseal-metaphyseal ends were fixed in 10% neutral buffered formalin at 4°C for 2 weeks, decalcified in 7% ethylene diamine tetraacetic acid (EDTA), infiltrated in JB4 medium, embedded in JB4 plastic, sectioned in the coronal plane at 5 micrometer thickness and stained with 1% toluidine blue. After decalcification, each bone was cut by scalpel in the mid-coronal plane. Each half was embedded for sectioning from midline to anterior regions and from midline to posterior regions. Multiple histologic sections were made in 50 bones, 26 cn/cn and 24 cn/+, spread throughout the age groups. i) Qualitative light microscopic assessments were made of the proximal humeral, distal femoral and proximal tibial epiphyses. ii) Histomorphometry. The region most affected in the cn/cn mutant was the hypertrophic zone of the physis which was invariably considerably diminished in extent compared with non-affected cn/+littermates. The Zidas (Zeiss Interactive Digitizing Analysis System, Zeiss, Germany) computerized histomorphometric system was used to quantify the ratio of the total area of the hypertrophic zone to the total area of the physis of the proximal humeral, distal femoral and proximal tibial physes. For each histologic section assessed, the entire physis from medial to lateral edges was included. Absolute area values were not assessed since, for comparative purposes, we could not make certain that the antero-posterior position of the section from one mouse physis corresponded to the position of another mouse physis. The percent relationship of the hypertrophic zone to the entire physis however, would be the same throughout. The physes (growth plates) were examined at 10 × magnification. A cursor outlined the entire growth plate (physeal) area from reserve zone to lower margin of hypertrophic zone at point of merger into metaphyseal region and then the area of the hypertrophic zone alone. The lower level of the hypertrophic zone is distinct; the hypertrophic zone was measured to the level of vascular and mesenchymal cell invasion into the lowermost hypertrophic cell columns. The upper level of the hypertrophic zone as it transforms from the proliferating cell layer is also reasonably distinct over a few cell thicknesses. One can also determine the beginning of the reserve cell layer although the line of demarcation in younger mice where epiphyseal cartilage persists must be carefully examined. All the histomorphometry measurements were done by one technician (LB) with levels repeatedly checked by the senior author (FS) for consistency. A ratio was calculated for each growth plate (cn/+ and cn/cn) section relating hypertrophic zone area to total physeal area. Multiple histologic sections were made from each of the physes. For each histologic section, histomorphometric measurements were made 3 times and a mean value was obtained. If there were 4 sections made from the same physis then 4 separate values were determined and the mean of those 4 indicated the single overall value for that physis. If 3 separate bones of the same physis (e.g., 3 cn/cn distal femurs from 3 mice of the same litter) at the same age were studied, then the mean value of the 3 represented the final measurement (for example of the distal femur cn/cn at 4 weeks of age). An average value was determined for each humeral, femoral and tibial physis at 3 time periods (2.5-3 weeks, 4–4.5 weeks, and 6–6.5 weeks). Comparisons were made between cn/cn and cn/+ratios: i) for all cn/cn physes compared to all cn/+physes (one value), ii) for each of the 3 physes (physis based) and iii) for each time period (age based).

### Transmission electron microscopy

Specimens for electron microscopy were obtained from 12 site and age-matched physes, 6 non-affected cn/+and 6 affected cn/cn. Tissue was studied from distal femur and proximal tibia at 2.5 weeks of age in 2 cn/+mice and 2 cn/cn mice, from distal femur at 4 weeks of age (1 cn/+, 1 cn/cn), from distal femur at 4.5 weeks of age (2 cn/+, 2 cn/cn), and from proximal humerus at 6.5 weeks of age (1 cn/+, 1 cn/cn). Epiphyseal regions were dissected into 1 mm^3^ segments immediately at sacrifice while immersed in 2.5% glutaraldehyde in a cold environment. Tissues were fixed in modified Karnovsky solution of 4% paraformaldehyde in 2.5% glutaraldehyde in 0.1 M cacodylate buffer, pH 7.4, for 3 hours in the cold prior to washing twice in 0.1 M cacodylate buffer. Tissues were decalcified in 7.5% EDTA in 2.5% glutaraldehyde, postfixed in 1% osmium tetroxide-sym collidine buffer, dehydrated in increasing concentrations of ethanol, infiltrated and embedded in Epon 812 resin (E.F. Fullam, Inc., Latham, NY), sectioned at 1 micrometer thickness, and stained with 1% toluidine blue for light microscopic study. Blocks chosen for ultrastructural analysis were trimmed, sectioned at 60 nm, stained with lead citrate and uranyl acetate, and examined at 60 kV on a Philips 300 transmission electron microscope. A total of 322 electron micrographs were examined encompassing similar numbers of assessments of cn/+and cn/cn tissue at the various ages.

## Results

### Gross appearance

The cn/cn mouse is smaller than the cn/+being approximately two-thirds the size. Size differential was present through the axial and appendicular skeleton including the tail and skull. Tails of the cn/cn mice were short but not twisted or curled (Figure 1). The skull was shortened and dome-shaped.

### Humeral, femoral and tibial length

The cn/cn long bones were invariably shorter than those of the non-affected cn/+littermates (Table 1). The mean cn/cn-cn/+length ratio encompassing all of the three bones at all time periods was 0.65 ± 0.03, n = 34, 17 ratios. The proportional shortness was similar in each of the three long bones studied: humerus 0.67 ± 0.002, n = 8; femur 0.62 ± 0.04, n = 4; and tibia 0.66 ± 0.01, n = 5; and did not change significantly at varying time periods: 2.5- 3 weeks 0.65 ± 0.02, n = 3; 4 – 4.5 weeks 0.64 ± 0.04, n = 7 and 6 – 6.5 weeks 0.67 ± 0.03, n = 7.

**Table 1:** Lengths (in millimeters) of cn/cn and cn/+bones*

Age (weeks)	Bone	cn/cn	cn/+	Length ratio (cn/cn)/(cn/+)
2.5	T	7.2	11.2	0.64
3	F	5.8	9.0	0.64
	T	7.2	10.6	0.68
4	H	6.0	9.0	0.67
	H	6.0	9.0	0.67
	H	6.5	9.5	0.68
	F	7.0	12.2	0.57
4.5	H	7.0	10.5	0.67
	F	7.2	12.0	0.60
	T	8.7	13.5	0.64
6	H	6.5	10.	0.65
	H	7.0	10.8	0.65
	H	7.0	11.0	0.64
6.5	H	8.0	11.0	0.73
	F	8.7	13.0	0.67
	T	10.3	15.7	0.66
	T	10.6	16.0	0.66
				**0.65 (±0.03, [N =17])**

*H* humerus; *F* femur; *T* tibia.

*measurements are from age-matched, side-matched littermates.

### Weights

The mean weight of 3 cn/cn mice at 4 weeks was 9.2 grams (r = 8.8-10) and of 3 cn/+littermates was 21.2 grams (r = 20.5-22.4), a ratio of 0.44. At 6 weeks the mean weight of 4 cn/cn mice was 17.1 grams (r = 15.8-18.9) and of 4 cn/+littermates 29.9 grams (r = 24.1-32.9), a ratio of 0.56.

### Radiographic studies

**Figure 2:**
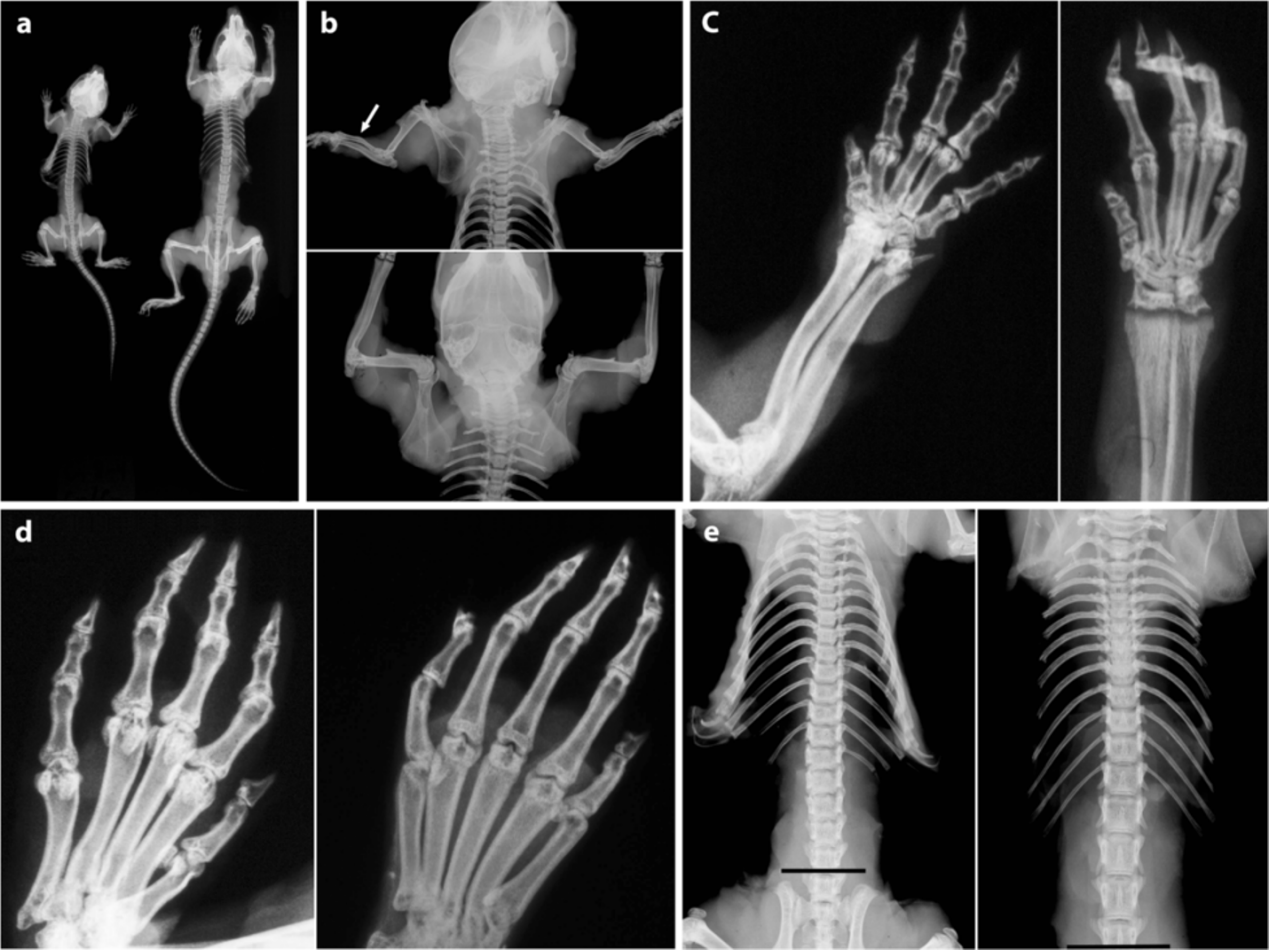
**Comparative Mouse Radiographs.** Comparative radiographs of cn/+ and cn/cn mice at 5 weeks from the same litter are shown. Pairs of mice were radiographed together for size comparison. **a**: Entire skeleton is shown with cn/+ (right) and cn/cn (left). Other than being smaller, vertebrae (cervical to sacral); ribs; pelvis; hip, knee, and shoulder joints; and tail vertebrae are normal in cn/cn compared to cn/+ regarding numbers, shape, and structure. **b**: Radiographs show cn/cn (top) and cn/+ (bottom) forelimbs. Humeri of both are normal. Note curved mid-diaphyseal radius bilaterally of cn/cn (arrow) compared to straight shafts of radius and ulna of cn/+. Shortening of cn/cn long bones is evident. Note normal rib and thoracic vertebral segmentation and structure of both mice. **c**: Radiographs show radius and ulna and metacarpals and phalanges of cn/+ (right) and cn/cn (left). Distal radial and ulnar growth plates are clearly visualized on both views **(b,c)** from cn/+ but are not seen due to distal deformity on both views in cn/cn. Metacarpals and phalanges are shorter in the cn/cn but are not otherwise deformed compared with cn/+. **d**: In dorsoventral views of metatarsals and phalanges [cn/cn (left), cn/+ (right)], other than shortness of the cn/cn bones, basic shape is the same and there are no bulbous deformities of cn/cn metatarsals or phalanges. **e**: Specimen radiographs of ribs and spine illustrate cn/+ (right) and cn/cn (left) littermates. Rib and vertebral segmentation is normal in both with the shorter length characterizing cn/cn. Shorter cn/cn ribs cause a narrower ribcage and chest. Interpedicular widths in mice normally decrease from L1 to L6, unlike in the human where they progressively widen. Radiographs are shown from the same cervical vertebrae above. Lines below at same position in the 4^th^ lumbar vertebrae demonstrate shorter stature of cn/cn mouse.

Radiographic studies of 2 cn/cn and 2 cn/+mice at 5 weeks of age from the same litter showed the cn/cn mutuant to be small but structurally similar to the normal cn/+littermate (Figure 2a). In the affected mutants there were 13 paired ribs which were normal in structure, the vertebrae from the upper cervical region to the tip of the tail were normally segmented and structurally unremarkable, and the number of spine and tail vertebrae was normal (7 cervical, 13 thoracic, 6 lumbar, 4 sacral, and 29 tail). The appendicular skeleton showed minimal differences other than shortness. The cn/cn long bone which deviated most from the cn/+normal was the radius with a definite anterior mid-diaphyseal bow (Figure 2b). The metacarpals, metatarsals and phalanges of upper and lower extremities were shorter than normal in the cn/cn but proportionate and structurally similar without an expanded bulbous shape (Figure 2c and d). The pelvis showed no evidence of a small sciatic notch or horizontal acetabulum; the hip, knee, and shoulder joints were normal; and the vertebrae and ribs were unremarkable in shape (Figure 2a,b, and e).

Linear measurements of several bones from the radiographs (other than humerus, femur and tibia) were made to determine additional length ratios at 5 weeks of age (Table 2). The interpedicular width at 2.5 weeks of age showed a decrease of 50% from the lumbar 1 vertebra to the lumbar 6 vertebra in both the cn/cn and cn/+littermates.

**Table 2:** Linear Measurements (in millimeters) from radiographs* at 5 weeks of age to assess length ratios

	cn/cn	cn/cn+	Measurements	Mean length ratio
Iliac crest to distal tip of ischium	11.1 (11.0-11.3)	16.8 (16.3-17.2)	8 per group	0.66
Radius Length				0.53
Ulna Length				0.59
Comparable metacarpal/metatarsal bones and phalanges (forelimb and hindlimb)				0.67

*measurements from radiographs accurate for determining length ratios but not actual lengths.

### Histology

**Figure 3:**
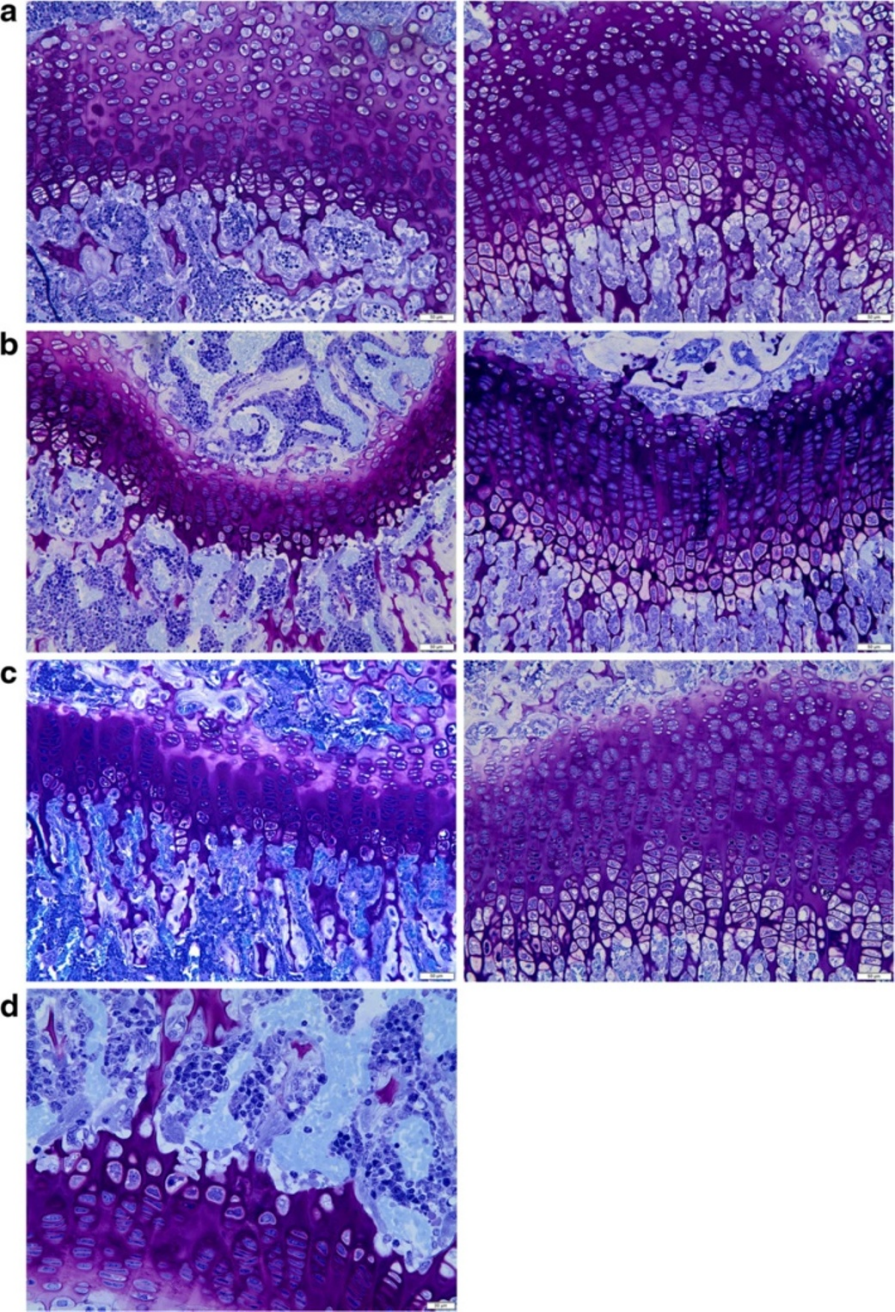
**Comparative Light Microscopy.** Light microscopic photomicrographs illustrate physeal/metaphyseal differences between cn/+ and cn/cn mice. **a-c**. Photomicrographs highlight the diminished hypertrophic zone of the physis in the cn/cn mouse (left) compared to the cn/+mouse (right) littermate. Images illustrate proximal humeral physes **(a)** at 2.5 weeks of age, distal femoral physes **(b)** at 3 weeks of age, and proximal tibial physes **(c)** at 5 weeks of age. The photomicrographs in the cn/cn mice also show a qualitative shortening of the proliferation zone of the physis even though the most prominent changes are in the hypertrophic zone. (×20, toluidine blue stain, marker = 50 μm). **d**. Photomicrograph shows prominent osteoclastic and vascular invasion activity at hypertrophic zone—metaphyseal junction in cn/cn mouse distal femur at 3 weeks. The vascular invasion however never advanced into the proliferating zone. (×40, toluidine blue stain, marker = 20 μm).

Light microscopic assessments of proximal humeral, distal femoral and proximal tibial epiphyses were performed in age matched cn/cn and cn/+siblings (Figure 3a-d). Epiphyseal, metaphyseal and diaphyseal shape was normal in the cn/cn compared to the cn/+littermates. The major abnormality in the cn/cn mice was in the epiphyseal growth plate (Figure 3a-d). The overall thickness of the physis appeared less in the cn/cn mice compared to age-matched, litter-matched controls. Resting and proliferating (palisading) zones were structurally normal although the proliferating zones were qualitatively somewhat shorter than expected, but the hypertrophic zone was markedly underdeveloped in each physis assessed at each time period (Figure 3a-c). In the cn/cn there were hypertrophic cells present but they were not always aligned vertically, the number was markedly less than in the normal, and the matrix was diminished and less well organized. In parts of some sections the hypertrophic zone was only 1–3 cells in length. In spite of this, vascular invasion of the hypertrophic zone occurred and metaphyseal bone formed. Vascular invasion from the metaphysis advanced close to the proliferating (palisading) zone but was still limited to the hypertrophic zone and accompanied by osteoclasts (technically chondroclasts since they were relating only to cartilage) resorbing the matrix (Figure 3d). Vascular/chondroclast presence within the proliferating (palisading) zone was not seen. The cortical bone of the intramembranous sequence appeared normal.

### Growth plate histomorphometry

In each physis assessed at each time period, the cumulative mean ratio (hypertrophic zone area/entire physeal area) in the affected cn/cn animals was always less than that of the corresponding physis of the non-affected cn/+littermate. Results from each specific physis at each specific time period were pooled to allow for a mean value of the ratio between the hypertrophic zone area and the entire growth plate area. Histomorphometric study involved 50 growth plates (cn/+24 and cn/cn 26) with 87 sections quantitated in cn/+bones and 117 in cn/cn bones (Table 3). When all findings were pooled for the respective cn/+and cn/cn groups, the ratio in cn/cn was 0.17 (±0.063) and in cn/+was 0.30 (±0.052). Assessments of values in each of the 3 bones and in each of the 3 age ranges showed similar findings (Table 4). The histomorphometric ratio quantifies the qualitative appearance of a diminished hypertrophic zone region in the affected mice.

**Table 3:** **Outline of growth plates and histologic sections quantitated**

	cn/cn			cn/+		
	Growth plates studied	# Histologic sections	Range of # of sections per growth plate	Growth plates studied	# Histologic sections	Range of # of sections per growth plate
Humerus	12	46	(1–9)	8	23	(2–5)
Femur	7	31	(1–7)	7	31	(2–11)
Tibia	7	40	(2–12)	9	30	(2–6)
	**26**	**117**		**24**	**87**	

# number.

**Table 4:** **Ratio of hypertrophic zone/entire physeal area in cn/cn and cn/+growth plates**

		cn/cn	cn/+
i) All bones (all time periods combined)		0.17 (±0.063)	0.30 (±0.052)
ii) Single bones (all time periods combined)	H	0.18 (±0.079)	0.29 (±0.065)
	F	0.17 (±0.049)	0.32 (±0.052)
	T	0.15 (±0.044)	0.29 (±0.037)
iii) Age-based ratios (all bones combined at specific age ranges in weeks)	2.5 -3	0.14 (±0.051)	0.30 (±0.041)
	4-4.5	0.20 (±0.066)	0.31 (±0.051)
	6-6.5	0.16 (±0.057)	0.29 (±0.059)

*H* proximal humerus; *F* distal femur; *T* proximal tibia.

### Ultrastructural findings

Growth plate ultrastructure of resting zone, proliferating (palisading) layers, hypertrophic zone and outer reaches of the metaphysis was assessed in affected cn/cn and normal cn/+littermates aged 2.5 (2 pairs), 4, 4.5 (2 pairs) and 6 weeks.

### cn/cn

**Figure 4:**
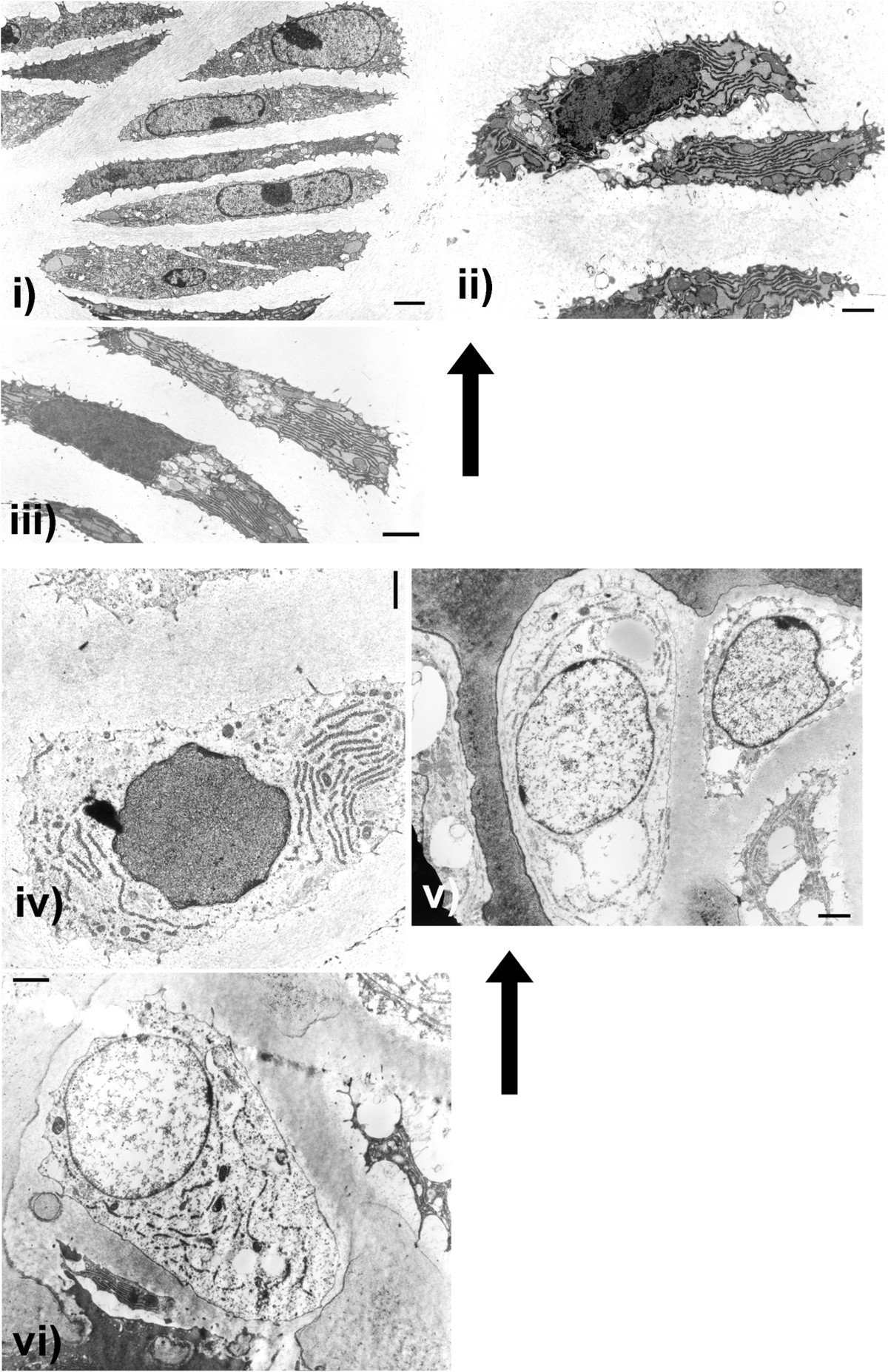
**Cn/cn Electron Microscopy.** Electron micrographs (EMs) illustrate chondrocyte sequential changes from upper proliferating (palisading) zone (i) to lower hypertrophic zone (vi) in cn/cn physes. Cn/cn hypertrophic zones were markedly shortened compared to cn/+ but sequential individual cell changes throughout physes were similar. Arrows point to cells closer to upper regions (proliferating zone) of physes with lower regions (hypertrophic zone) below. EMs from cn/cn distal femoral and proximal tibial physes at 2.5 and 4.5 weeks are shown; proliferating zone (i to iii) and progressively lower cells in hypertrophic zone (iv to vi). Upper proliferating cell layers (i, ii and ii) show active flattened chondrocytes with abundant, well organized rough endoplasmic reticulum (RER) with dilated cisternae containing a homogeneous material indicative of active protein synthesis. In the cn/cn chondrocytes there were neither massively dilated collections of RER or abnormal electron dense collections within the RER which, if present, would indicate an ER storage disease. The hypertrophic cells in iv-vi show progressive hypertrophy, diminution of RER organelles but with some RER present even at the lowest levels at the metaphyseal junction. The hypertrophic cells in both sequences show well preserved organelles with most cells filling their lacunae with cell membranes intact. Line markers: i) 2.20 μm; ii) 1.00 μm; iii) 1.55 μm; iv) 1.26 μm; v) 1.55 μm and vi) 1.55 μm.

The cn/cn findings are illustrated in Figure 4 i-iii showing chondrocytes from the proliferating (palisading) layers and iv-vi from the hypertrophic zone. The proliferating zone appeared normal in the cn/cn mutants. Cells were stacked vertically with clear definition of transverse and longitudinal septae, nuclei were normally shaped and positioned, Golgi regions were well developed and there was abundant rough endoplasmic reticulum with dilated cisternae filled with a moderately electron-dense homogeneous material (Figure 4 i-iii). Surrounding cartilage matrix was ultrastructurally normal even at 50,000 times magnification. Individual hypertrophic chondrocytes were unremarkable although at low power the total area of hypertrophic cell formation was markedly diminished (Figure 4 iv-vi). In hypertrophic cells approaching the metaphysis, there were progressively more spaces empty of organelles but with some persisting dilated circular and linear collections of rough endoplasmic reticulum as well as round nuclei. No inclusion bodies were seen in cytoplasm or nuclei throughout all layers. Intact functioning hypertrophic chondrocytes persisted even within and at lower parts of the markedly shortened hypertrophic zones as evidenced by cells filling their lacunae with cell membranes intact (Figure 4 iv, v, and vi), round nuclei (Figure 4 v and vi), and well-structured rough endoplasmic reticulum (RER) (Figure 4 iv-vi). Intracellular glycogen was not prominent in any specimens. Vascular invasion of hypertrophic cell lacunae along with undifferentiated mesenchymal cells and associated new bone formation on cartilage cores was normal.

### cn/+

**Figure 5:**
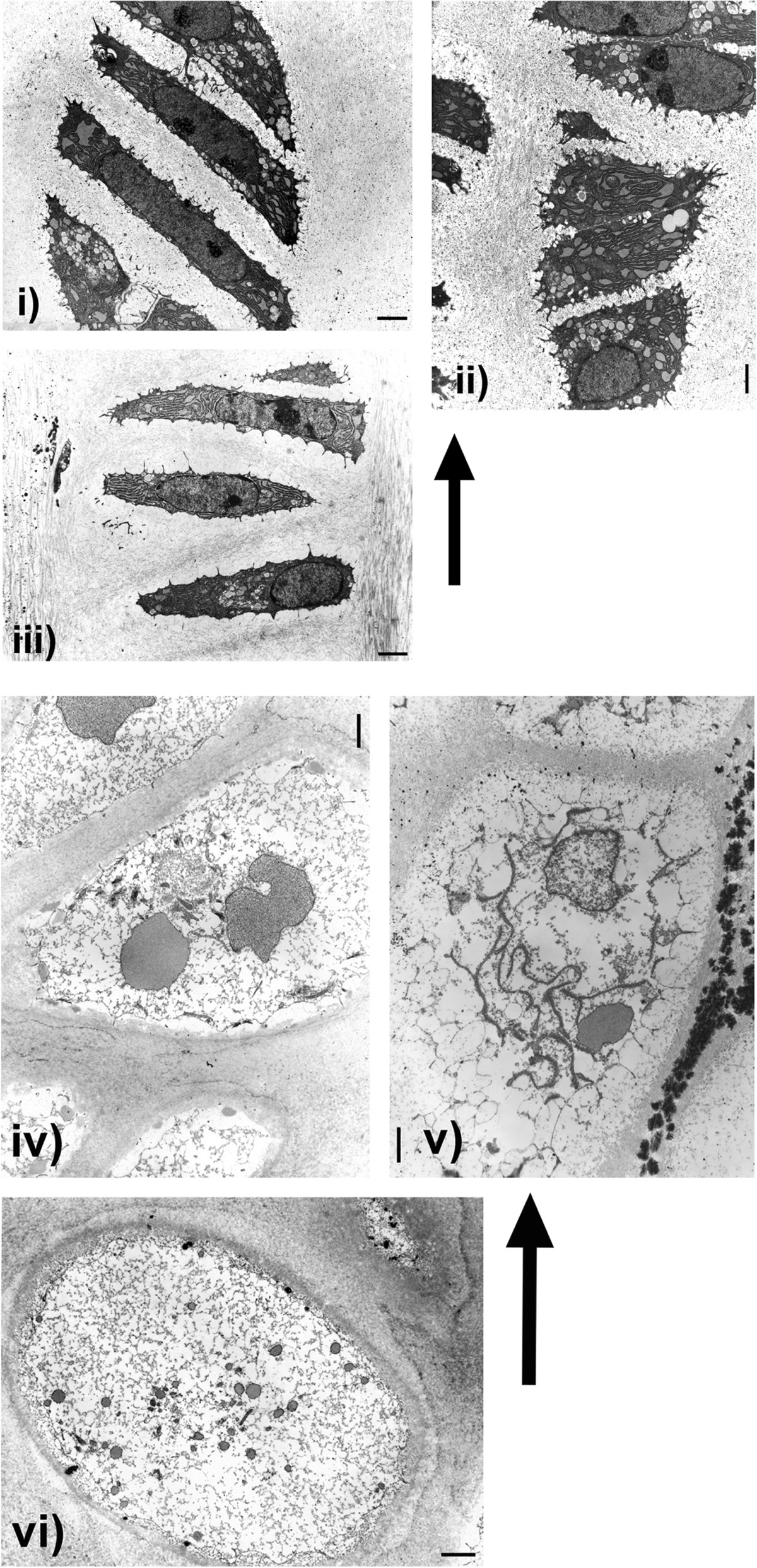
**Cn/+ Electron Microscopy.** Images from the normal cn/+ physes from site and age-matched littermates, in relation to those seen in Figure 4, are shown. Upper proliferating cells (i-iii) show flattened chondrocytes with abundant well organized rough endoplasmic reticulum (RER) with dilated cisternae containing a homogenous material indicative of active protein synthesis. The hypertrophic cells (iv-vi) show progressively diminishing RER organelles but these are still present even at the lowest levels at the metaphyseal junction. Arrows point in direction of cells closer to the upper regions of the respective zones. 5v shows persistent mineralization of a longitudinal cartilage septum. Line markings: i) 2.20 μm; ii) 1.55 μm; iii) 2.20 μm; iv) 2.20 μm; v) 2.20 μm; and vi) 2.20 μm.

The cn/+physes are shown in Figure 5 i-iii and iv-vi. The proliferating zone region showed orderly stacked layers of chondrocytes with abundant rough endoplasmic reticulum with dilated cisternae containing a moderately electron-dense homogenous material (Figure 5 i-iii). Golgi apparatuses were abundant. Occasional cells contained glycogen in physiologic amounts. There was an orderly longitudinal orientation of collagen fibers in the columnar matrix (Figure 5 iii). In the hypertrophic zone the characteristic diminution of organelles within the enlarged cytoplasmic mass was seen in cells closer towards the metaphysis (Figure 5 iv-vi). Isolated collections of well-formed linear rough endoplasmic reticulum with some dilated regions persisted even at the lowermost cells adjacent to the metaphysis (Figure 5 v and vi). At the lowermost parts there were dilated cisternae of rough endoplasmic reticulum in areas otherwise empty of organelles. Many hypertrophic cells continued to fill their lacunae with cell membranes intact immediately adjacent to the matrix (Figure 5 iv and vi). There was vascular invasion of hypertrophic cell lacunae with undifferentiated mesenchymal cells and new bone formation on cartilage cores.

## Discussion

### Short stature distributed throughout the skeleton

In affected cn/cn mutants shortening involves skull, axial skeleton and tail, and limbs. Affected long bones are two-thirds the length of their normal littermates with each of humerus, femur and tibia showing the same proportions as well as continuing similarity in length proportion from 2.5 to 6.5 weeks (Table 1). Comparative measurements from skeletal radiographs also show similar size ratios of cn/cn to cn/+bones in the pelvis and metacarpals, metatarsals, and corresponding phalanges (Table 2). The spine length ratio was 0.58. In our radiographs, the radius was marginally shorter than the ulna (0.53/0.59) and both were slightly shorter than the more proximal humerus. In the original description of the cn/cn mutants the length ratios were similar to those documented in this study; humeral length ratio was 0.66, femoral 0.61 and tibial 0.60 with shorter spine cn/cn length 0.58, radius 0.63, ulna 0.62, and pelvis 0.61 [1]. Similar values were measured in other studies [2, 3]. Shortening in cn/cn mutants affects both axial and appendicular portions of the body and proximal, middle, and distal parts of each limb. The shortening is not disproportionate as that term is used in the human skeletal dysplasia literature when the upper and lower extremities are markedly more affected than vertebral column axial structures (short limb dwarfism) or vice versa (short trunk dwarfism). Cn/cn mice also have decreased skull growth concentrated at the spheno-occipital and mid-sphenoidal synchondroses, nasal septal cartilage and condylar cartilage [5, 18].

### Bone shape

The overall shape of the bones is normal with the major exception of the radius which has a distinct mid-diaphyseal bowing. This appears to interfere distally with the carpal relationship due to slightly greater radial than ulnar shortness. This specific radial deformity has not been noted before in the cn/cn mouse (1–13). Other than the shortness, however radiographs show no evident shape abnormalities of metacarpals, metatarsals, and corresponding phalanges.

### Histology

The primary area of abnormality of the growth plate by light microscopy involves the markedly shortened hypertrophic zone. Our light microscopic study, like earlier studies, clearly defines deficiency in the hypertrophic zone chondrocytes with a qualitative suggestion of slight shortening of the proliferative zone as well. Some studies have documented a slight decrease in size of the proliferating zone along with the much more affected hypertrophic zone [7, 9, 10]. Growth in length is a function of i) cell proliferation in the upper proliferating (palisading) zone, ii) cartilage matrix synthesis primarily within the lower palisading zone and iii) chondrocyte hypertrophy in the hypertrophic zone. While structural abnormalities are primarily within the markedly shortened hypertrophic zone, it is not completely absent and still is associated with vascular and osteoclastic invasion from the metaphysis and bone formation on cartilage cores.

### Histomorphometry

The overall area of the hypertrophic zone in relation to the entire growth plate shows a ratio of 0.17 (±0.063) in affected mutants and 0.30 (±0.052) in non-affected littermates. In a study of the kinetics of growth cartilage in the cn/cn mouse, Thurston and colleagues also documented the reduced hypertrophic cell height in the proximal tibia in mutants aged 16 and 17 days [8] but some were more affected than others with slightly reduced labeling index in the proliferative zone in the most severe group. Previous studies have invariably noted that the hypertrophic cell region is the most markedly affected region of the physis in the cn/cn mouse [3, 4, 6, 7, 9, 10].

### Physeal ultrastructure

Before this study, ultrastructural studies of cn/cn physes have not been done in detail. We show a normal sequence of change in the hypertrophic zone even though the number of hypertrophic chondrocytes is less and the zone is markedly shortened. The only previous ultrastructural study of cn/cn physes described abnormally large cytoplasmic deposits of glycogen in maturing (proliferating zone) and hypertrophic chondrocytes which appeared to increase with age but otherwise concluded that the fine structure of cartilage and bone cells in the cn/cn mice differed very little from normal [19]. We did not find a pathologic increase of glycogen in physeal chondrocytes at any level. Ultrastructural assessment of articular cartilage chondrocytes of affected cn/cn mutants in another study was unremarkable compared to normal littermates at 3 weeks but then developed deposition of glycogen and premature degeneration [20]. Assessments by Wilkstrom et al. [9] and Bonucci et al. [19] showed that calcification of the cartilage matrix took place in cn/cn mice normally with mediation by matrix vesicles. Normal metaphyseal zone mineralization was noted even in mutant mice with marked hypertrophic zone underdevelopment.

We demonstrate that the cn/cn cells pass through the normal sequence of ultrastructural changes with no evidence of an endoplasmic reticulum storage disorder in cn/cn chondrocytes. The findings are consistent with an abnormality primarily affecting the hypertrophic chondrocyte region rather than matrix synthesis. Assessment in the cn/cn growth plates in this study showed no ultrastructural evidence of an endoplasmic reticulum storage disorder. Endoplasmic reticulum storage diseases (ERSD) (or ERAD-endoplasmic reticulum associated protein degradation) were defined initially in other skeletal dysplasias by abnormal chondrocyte ultrastructure [21–23]. Examples include: i) prominent RER inclusions in morphologically distinct whorled patterns characterizing chondrocytes in pseudoachondroplasia, [24, 25]; dilated RER in physeal and epiphyseal chondrocytes showing abnormal electron-dense accumulations randomly oriented and diffusely marginated progressing to well-marginated collections of wavy rod-like structures with a circular orientation parallel to the outer edges of the RER in spondyloepimetaphyseal dysplasia with scoliosis [26]; and dilated RER containing linear lamellae of alternating electron-dense and electron-lucent material in chondrocytes from epiphyseal cartilage in multiple epiphyseal dysplasia with mild myopathy due to a mutation in the α-3 chain of type IX collagen [27]. While some consider massively dilated RER in chondrocytes containing a homogenous material to represent an ERSD, the dilated RER in proliferating cell chondrocytes in both cn/cn and cn/+mice was similar in this study.

### Similar and dissimilar structural findings in mouse cn/cn mutant corresponding to human AMDM

The cn/cn mouse has the same molecular defect as the human skeletal dysplasia, acromesomelic dysplasia Maroteaux type (AMDM) [14]. A study of 115 cn/cn mice using linkage analysis mapped the cn locus to chromosome 4 with the natriuretic peptide receptor 2 (Npr2) gene as the primary candidate for the cn mutation. This gene encodes a receptor for C-type natriuretic peptide (CNP) that positively regulates longitudinal bone growth by producing a CNP in response to CNP binding to the extracellular domain. Hume et al. note defective cellular trafficking in many human missense NPR-B mutations underlying AMDM [28]. At least 28 different mutations in the NPR-2 gene have been identified. They generated all the missense mutations found in AMDM patients and cn/cn mice and examined their subcellular localization in cultured cells by confocal microscopy. They felt that 11 of 12 mutants were retained in the ER. Our study did not find ultrastructural evidence for this in the cn/cn mouse but the sensitivity of the modalities clearly differs.

The CNP/NPR-B pathway helps regulate bone formation through the endochondral pathway and mice with targeted disruption of CNP subsequently develop severe dwarfism due to impaired endochondral ossification [29]. The Nppc −/− mice had narrowed growth plates compared with Nppc +/+ with the heights of the proliferative and hypertrophic zones markedly reduced with no differences in the resting zones. Mice generated to lack the guanyl cyclase B (GC-B) receptor (for the C-type natriuretic peptide) show considerable impairment of endochondral ossification and decreased vertebral and limb-bone growth [30]. Hypertrophic chondrocytes are markedly diminished compared to the proximal cell layers. Mice lacking CNP (Nppc −/−) exhibit a skeletal dwarfism with the same growth plate histology as NPR-2 −/−. CNP increases the number of chondrogenic condensations of mouse embryonic limb bud cells in micromass culture and increases expression of enzymes involved in chondroitin sulfate synthesis and ultimately cartilage glycosaminoglycans [31].

Approximately one in 30 individuals with currently idiopathic short stature is a carrier of NPR2 mutations [32]. NPR-2 the natriuretic peptide receptor B guanyl cyclase GC-B gene has been identified as responsible for causing AMDM; it is a receptor for C-type natriuretic peptide (CNP) that acts locally as a paracrine and /or autocrine regulator in many tissues including bone [33]. NPR-2 (NPR-B) was contained in the interval on chromosome 9 where 18 families with AMDM mapped. Mutations causing AMDM have been found in the extracellular ligand binding domain and transmembrane domain as well as the intracellular kinase homology and guanyl cyclase domains. The role of CNP in regulating endochondral ossification is being increasingly outlined [34–36].

With the same gene mutation affecting both the cn/cn mouse and human short stature skeletal dysplasia patients with AMDM, the changes in the mouse begin to demonstrate how molecular abnormalities in the C-type natriuretic peptide gene translate to structural abnormalities. We demonstrate that some of the primary localizing radiographic changes noted in the human disorder are also seen in the cn/cn mouse. As well as short stature, the radius and ulna abnormalities especially the shortened bowed radius in cn/cn mice are strikingly similar to findings in human AMDM [37–40]. There are also however radiographic differences with the apparent same mutation in the cn/cn mouse and human AMDM skeletons. In the human involvement is clearly disproportionate with the extremities more affected than the axial skeleton and the upper extremities in particular showing marked forearm, wrist and hand shortening [37–40] (as indicated by the term acromesomelic). In the cn/cn mouse, the shortening tends to be more uniform throughout. Shortening and bony deformation in human AMDM are particularly evident in the forearm, hand and foot but while shortening and radial deformation are seen in cn/cn mice, the widened bulbous appearances of human metacarpals/metatarsals and phalanges are not seen (Figure 2c,d). Human vertebral changes, such as vertebral body wedging leading to a loss of thoracic kyphosis and occasional lumbar kyphosis, are not seen in the mouse. Nguyen and Xu have outlined the values of studies in corresponding mouse and human mutations [41]. It remains to be seen whether phenotypic mouse/human differences are due to size and complexity variations or whether additional gene abnormalities exist.

The hypertrophic chondrocyte zone is a region of continuing structural and molecular study [42–44]. It will be important to assess further how the hypertrophic zonal underdevelopment as seen in the cn/cn mouse is caused by the NPR-2 gene abnormalities. Efforts are also underway to investigate how translational research can adapt the stimulating effects of the C-type natriuretic peptide on endochondral bone growth into therapies for skeletal dysplasias [45].

## Conclusion

The gene abnormality in the cn/cn mouse is a loss-of-function mutation in the natriuretic peptide receptor 2 (NPR-2), the same abnormality detected in the human skeletal dysplasia, acromesomelic dysplasia Maroteaux type (AMDM). Electron microscopic studies of the physes in the cn/cn and cn/+mice assess chondrocyte findings for the first time and show no structural abnormalities. Histology and histomorphometry show specific underdevelopment of the hypertrophic zone of the physes. We identify radiographic deformities in the radius of the mouse cn/cn mutant (bowing of the mid-diaphyseal region) not described previously and similar to those in human AMDM. This study enables a clearer understanding of the locali-zation and mechanism of growth abnormality with this mutation with implications for both the mouse and human disorders.

## Authors’ original submitted files for images

Below are the links to the authors’ original submitted files for images. Authors’ original file for figure 1 Authors’ original file for figure 2 Authors’ original file for figure 3 Authors’ original file for figure 4 Authors’ original file for figure 5 Authors’ original file for figure 6 Authors’ original file for figure 7 Authors’ original file for figure 8 Authors’ original file for figure 9 Authors’ original file for figure 10 Authors’ original file for figure 11 Authors’ original file for figure 12

## Abbreviations

NPR-2 (NPR-B): Natriuretic peptide receptor 2
CNP: C-type natriuretic peptide
AMDM: Acromesomelic Dysplasia Maroteaux
EDTA: Ethylene diamine tetraacetic acid
ERSD: Endoplasmic reticulum storage disease
ERAD: Endoplasmic reticulum associated protein degradation
RER: Rough endoplasmic reticulum
GC-B: Guanylyl cyclase B.
