# Supplementary figures and images for: The cn/cn dwarf mouse. Histomorphometric, ultrastructural, and radiographic study in mutants corresponding to human acromesomelic dysplasia Maroteaux type (AMDM)
Source: BMC Musculoskelet Disord. 2014 Oct 15;15:347. doi: 10.1186/1471-2474-15-347 (PMC4219045; doi:10.1186/1471-2474-15-347)

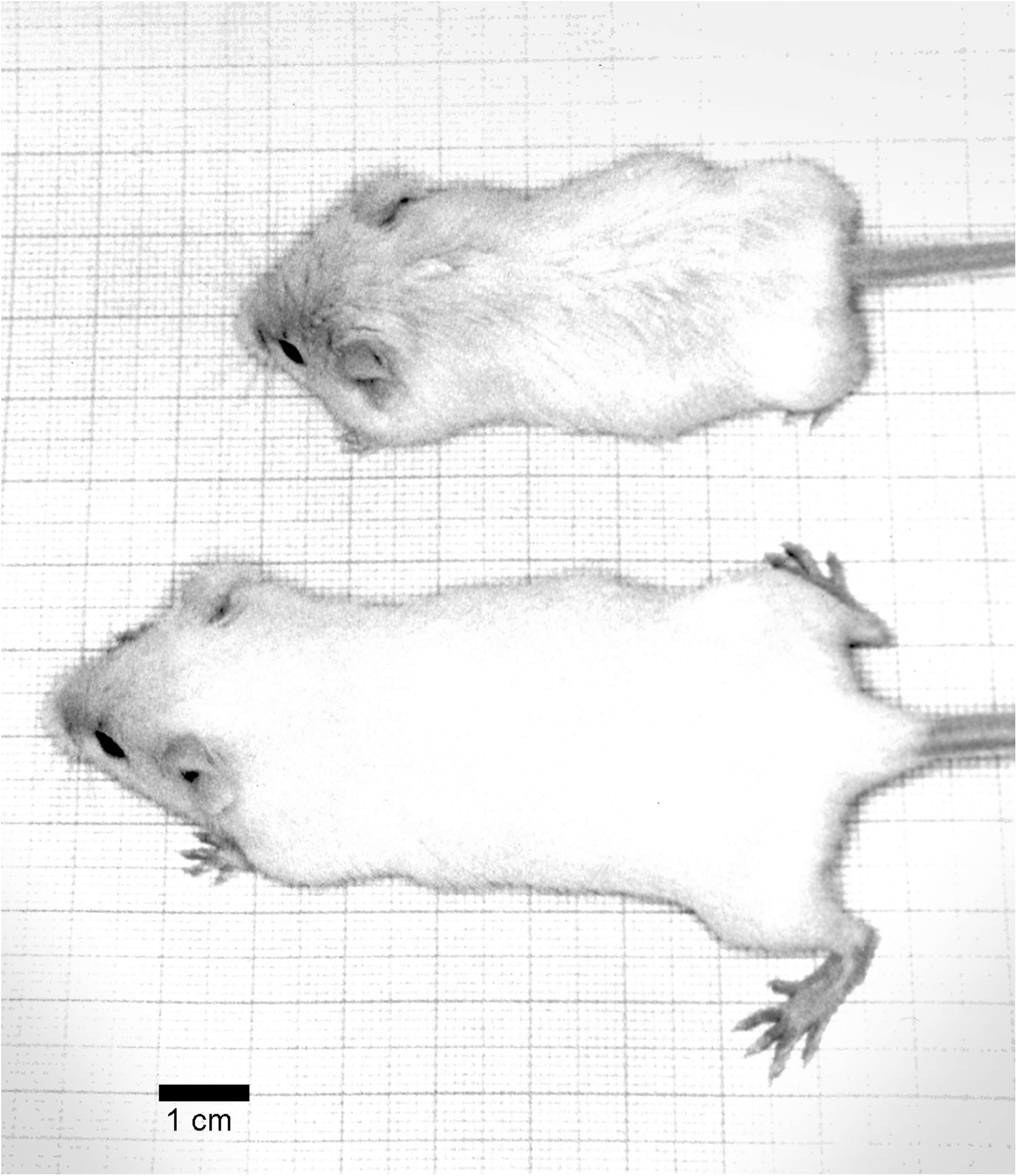

Supplement: Supplementary file 1 — Authors’ original file for figure 1 [file 12891_2014_2291_MOESM1_ESM.tiff]

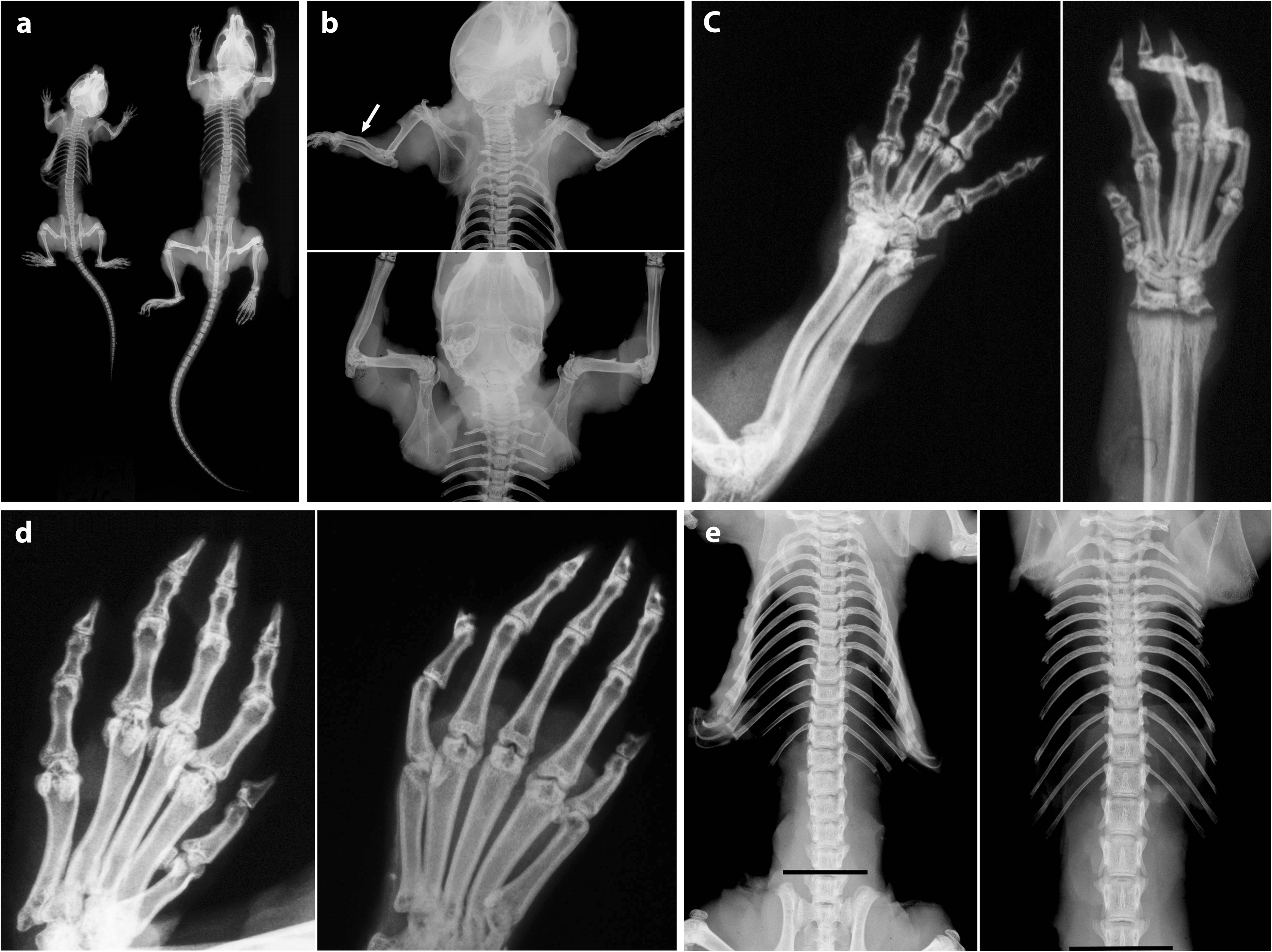

Supplement: Supplementary file 2 — Authors’ original file for figure 2 [file 12891_2014_2291_MOESM2_ESM.tiff]

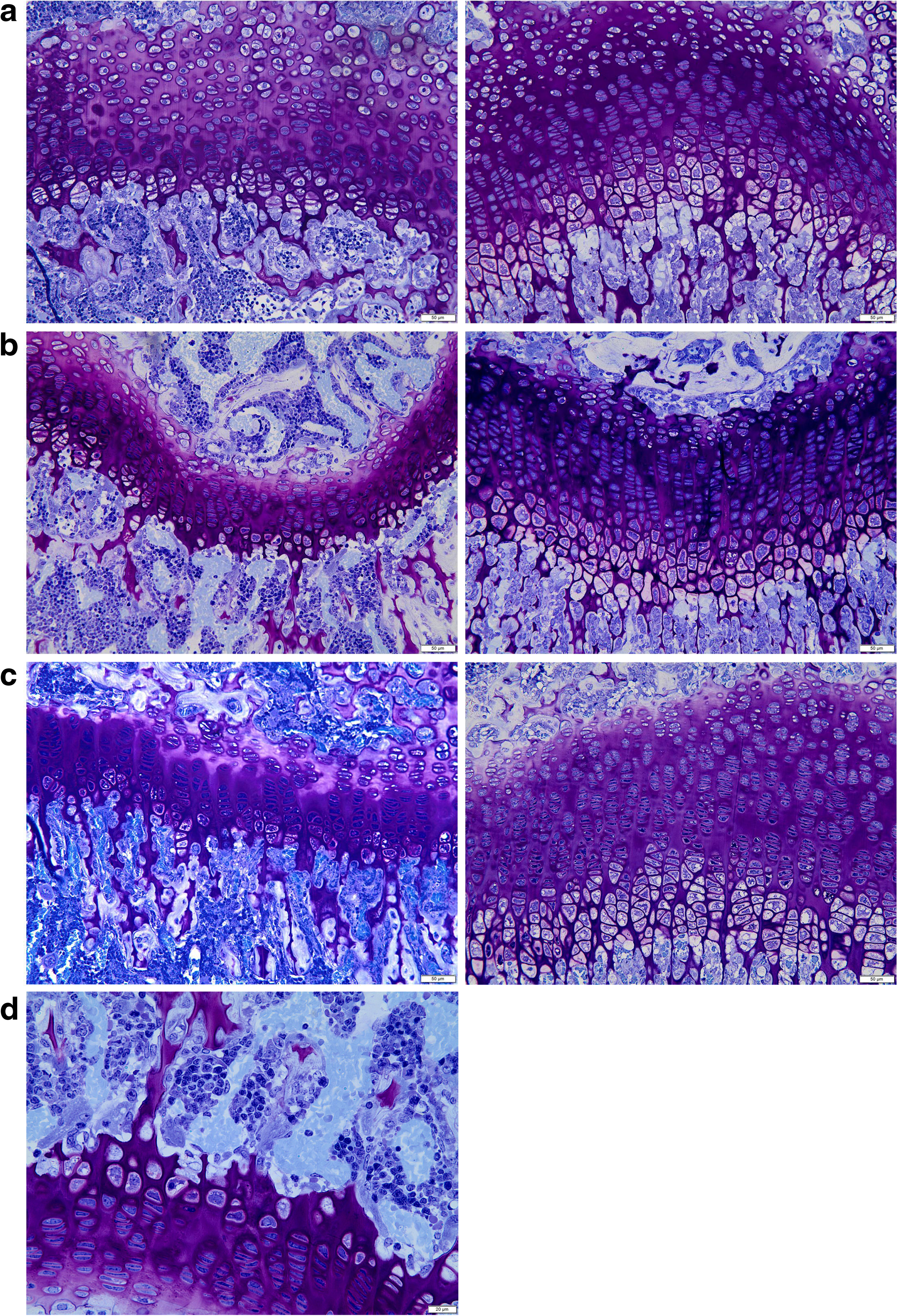

Supplement: Supplementary file 3 — Authors’ original file for figure 3 [file 12891_2014_2291_MOESM3_ESM.tiff]

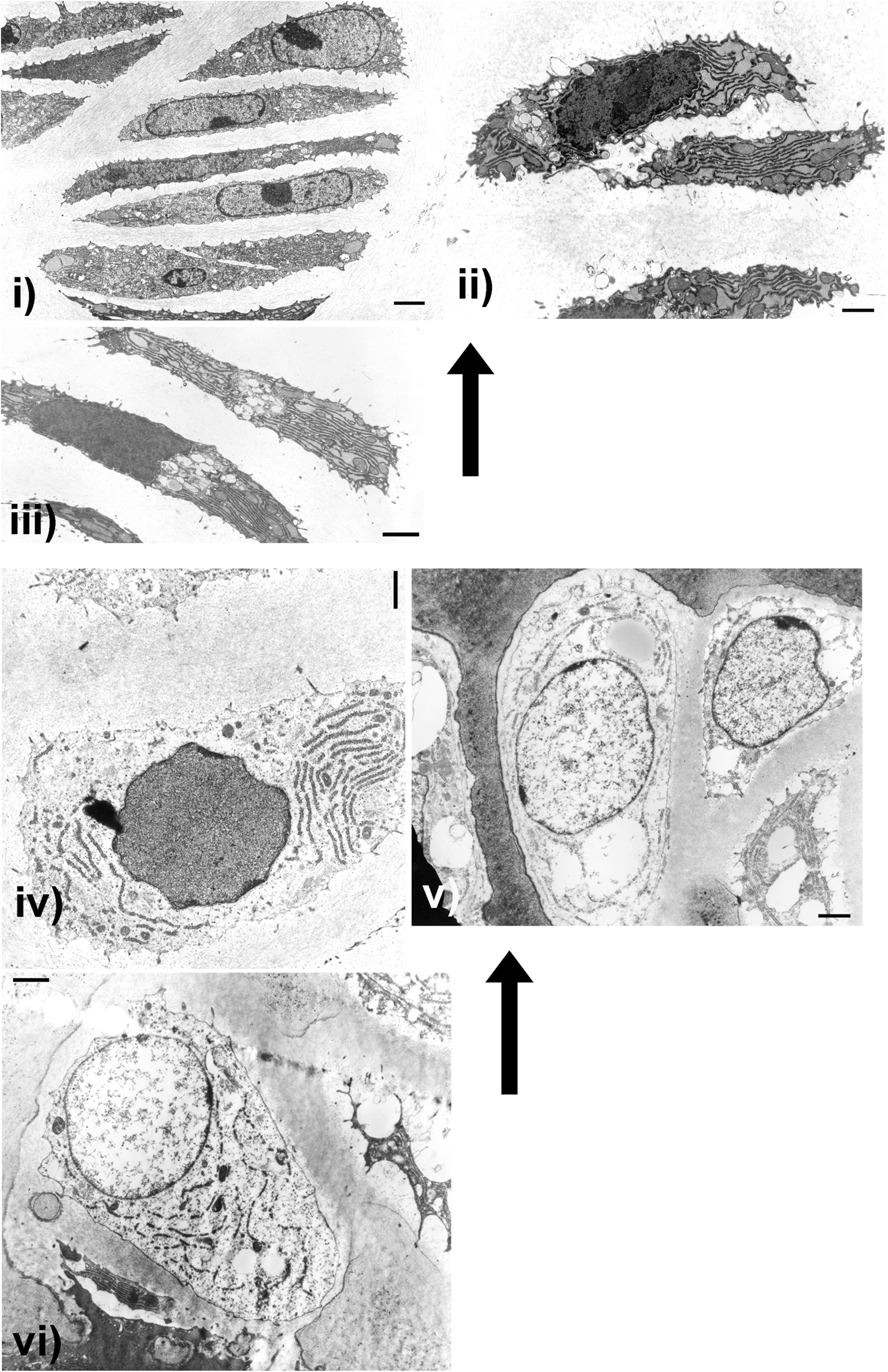

Supplement: Supplementary file 4 — Authors’ original file for figure 4 [file 12891_2014_2291_MOESM4_ESM.tiff]

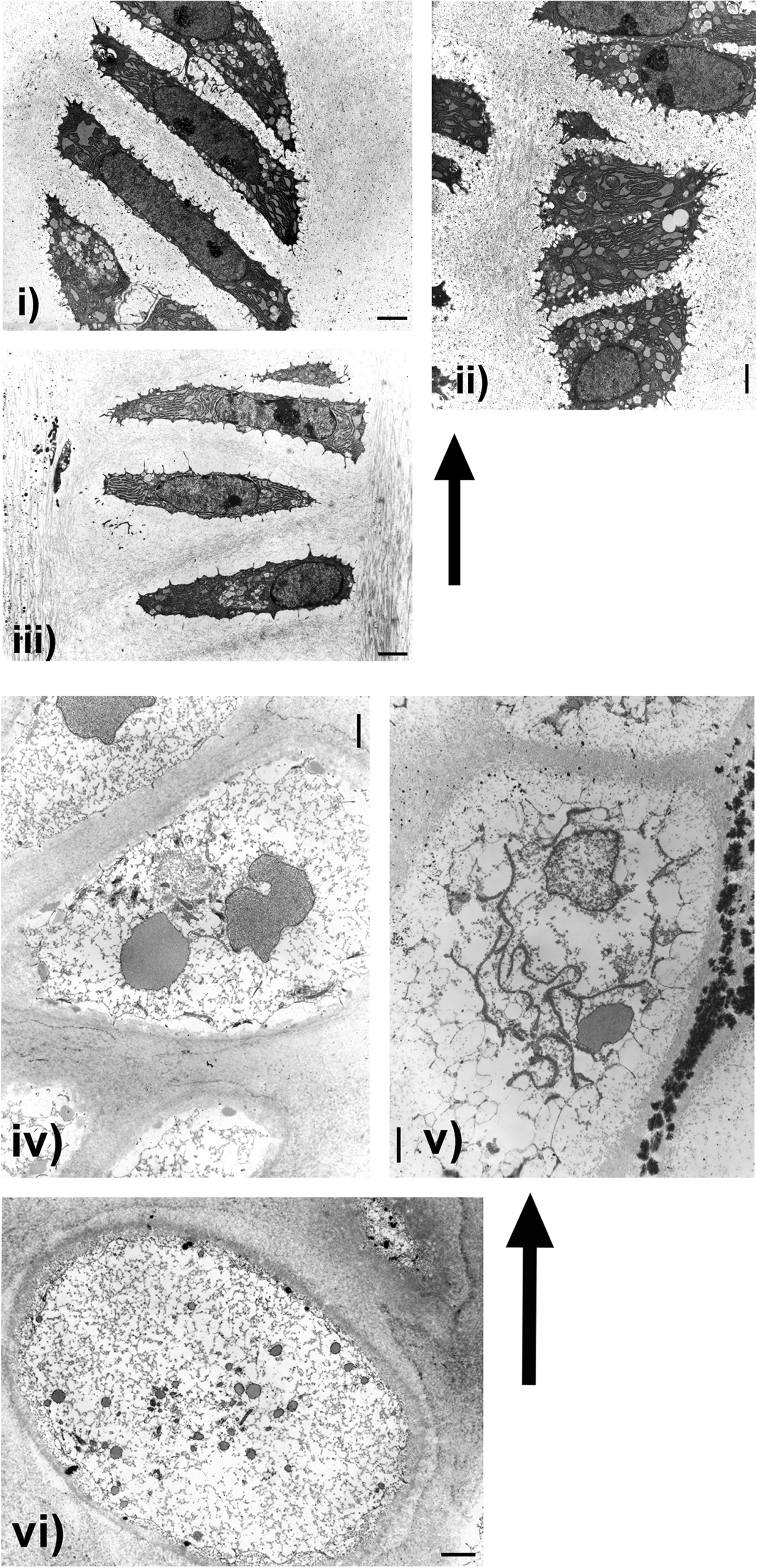

Supplement: Supplementary file 5 — Authors’ original file for figure 5 [file 12891_2014_2291_MOESM5_ESM.tiff]

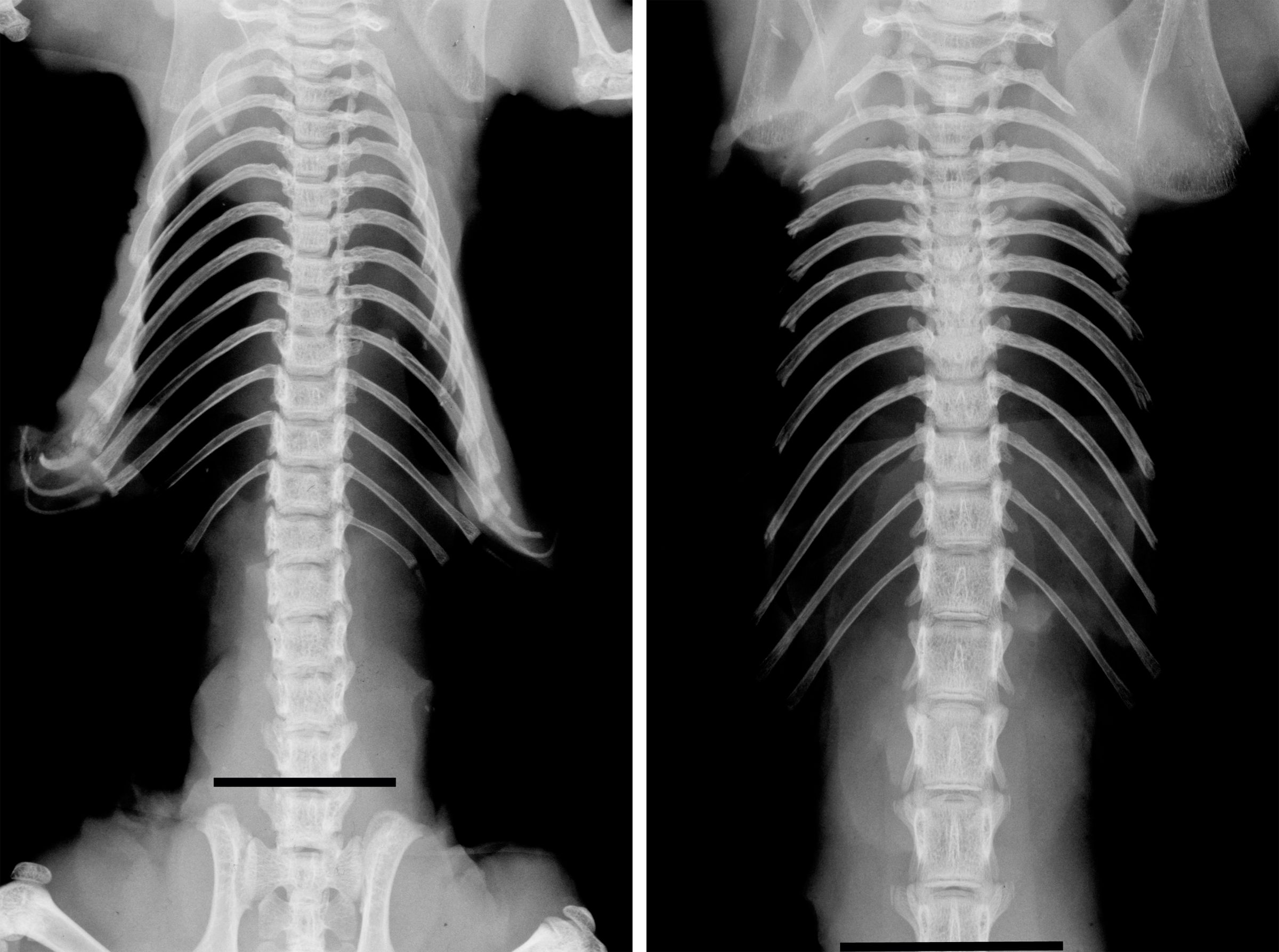

Supplement: Supplementary file 6 — Authors’ original file for figure 6 [file 12891_2014_2291_MOESM6_ESM.jpeg]

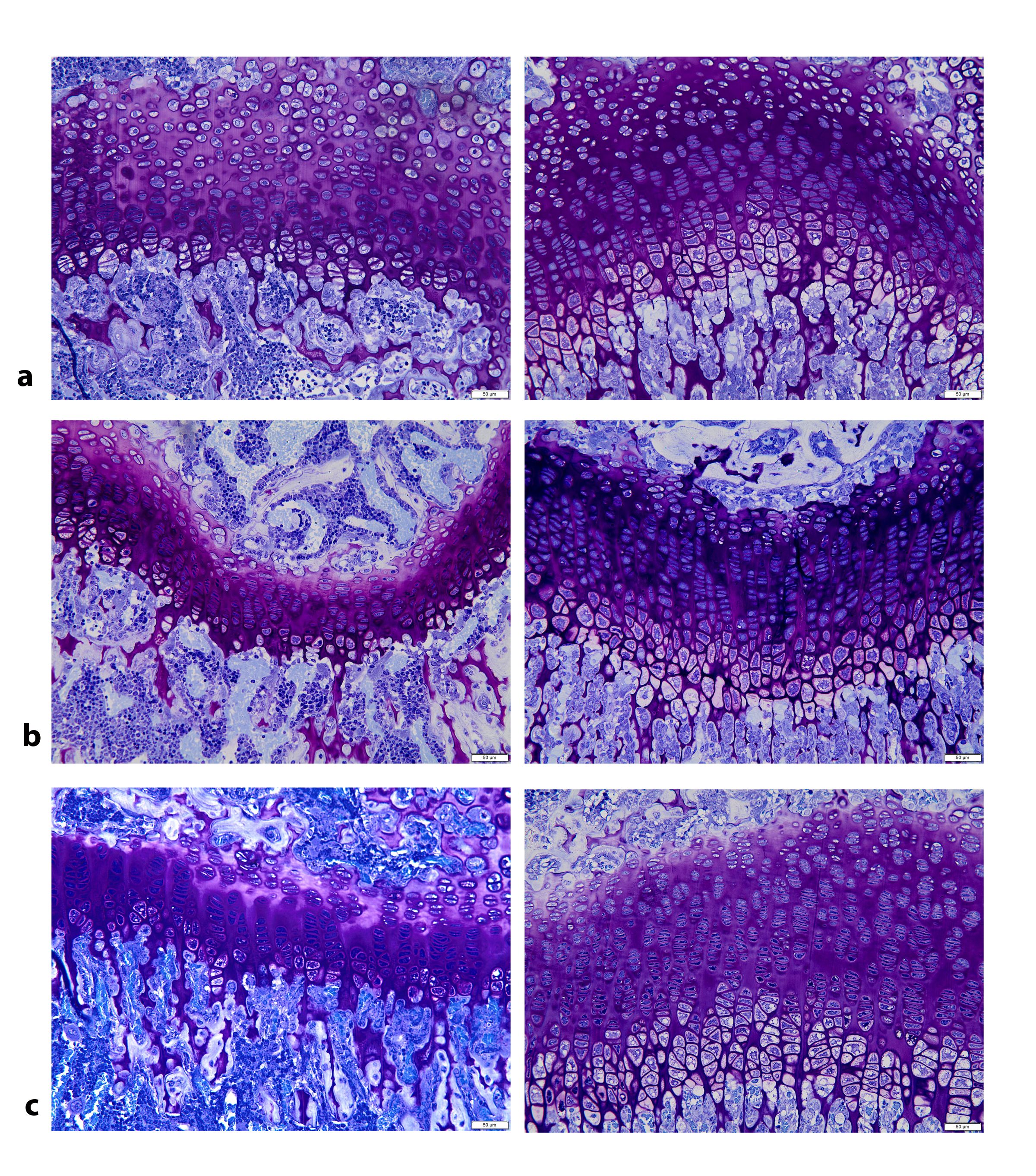

Supplement: Supplementary file 7 — Authors’ original file for figure 7 [file 12891_2014_2291_MOESM7_ESM.jpeg]

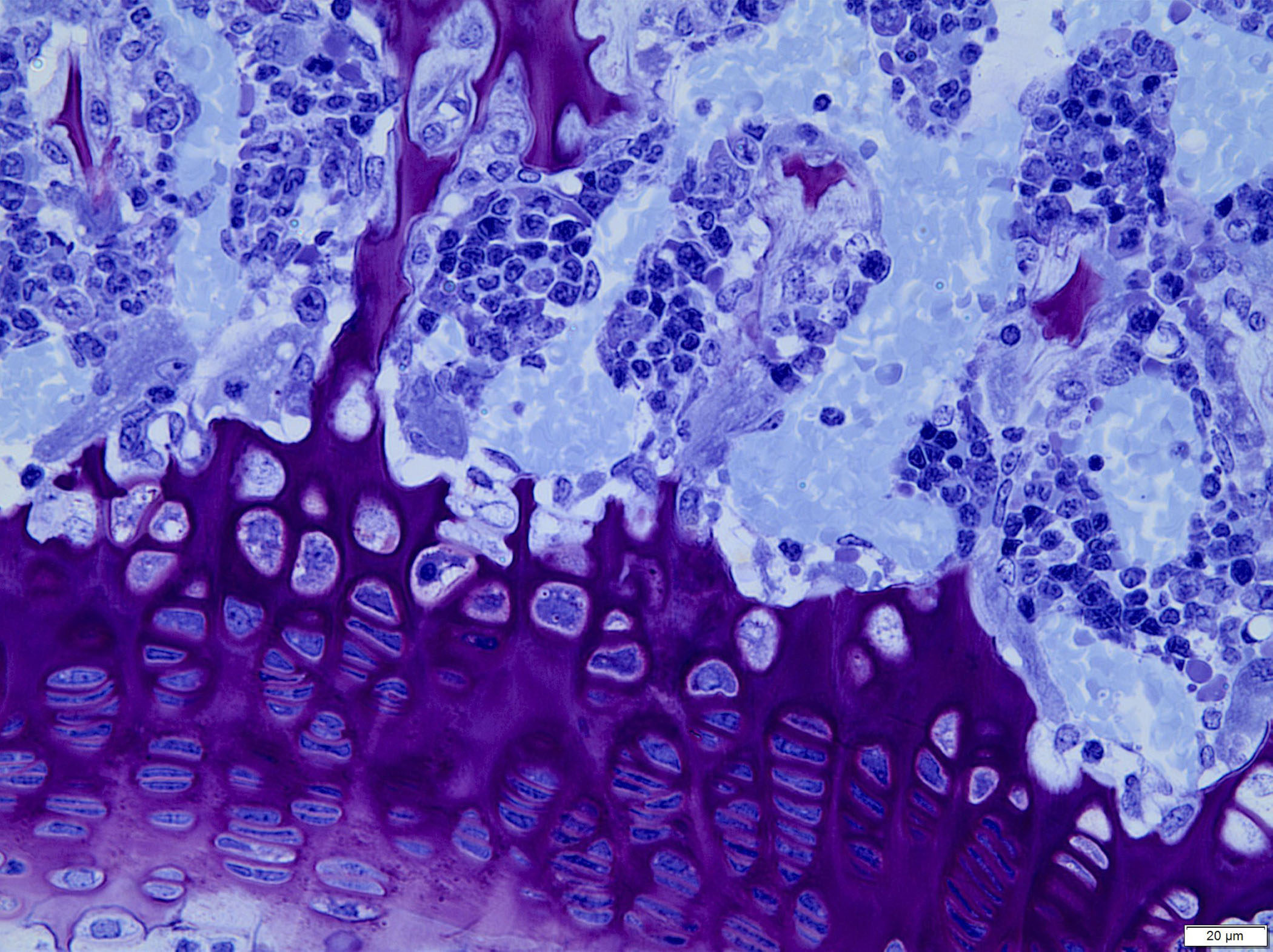

Supplement: Supplementary file 8 — Authors’ original file for figure 8 [file 12891_2014_2291_MOESM8_ESM.jpeg]

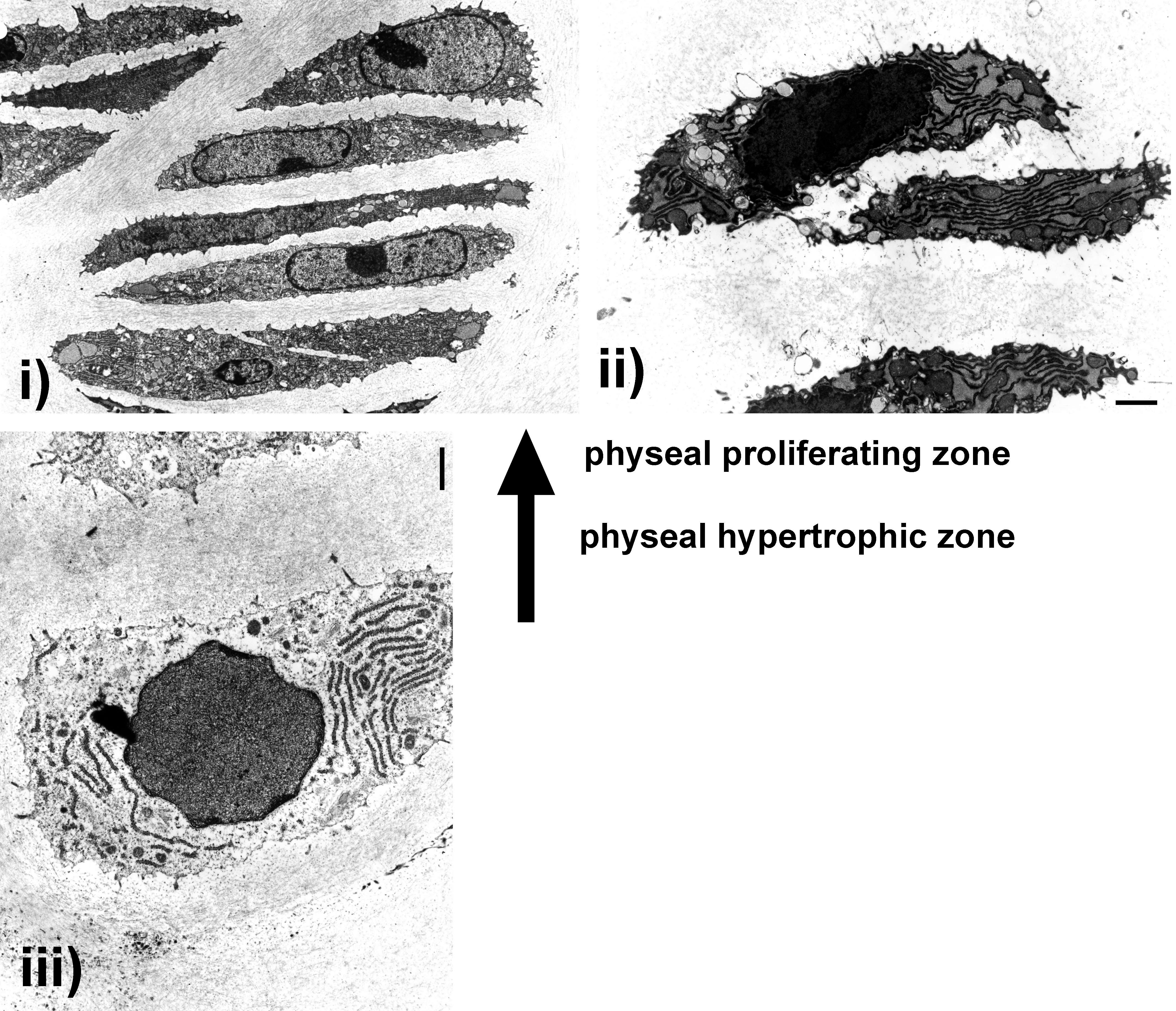

Supplement: Supplementary file 9 — Authors’ original file for figure 9 [file 12891_2014_2291_MOESM9_ESM.jpeg]

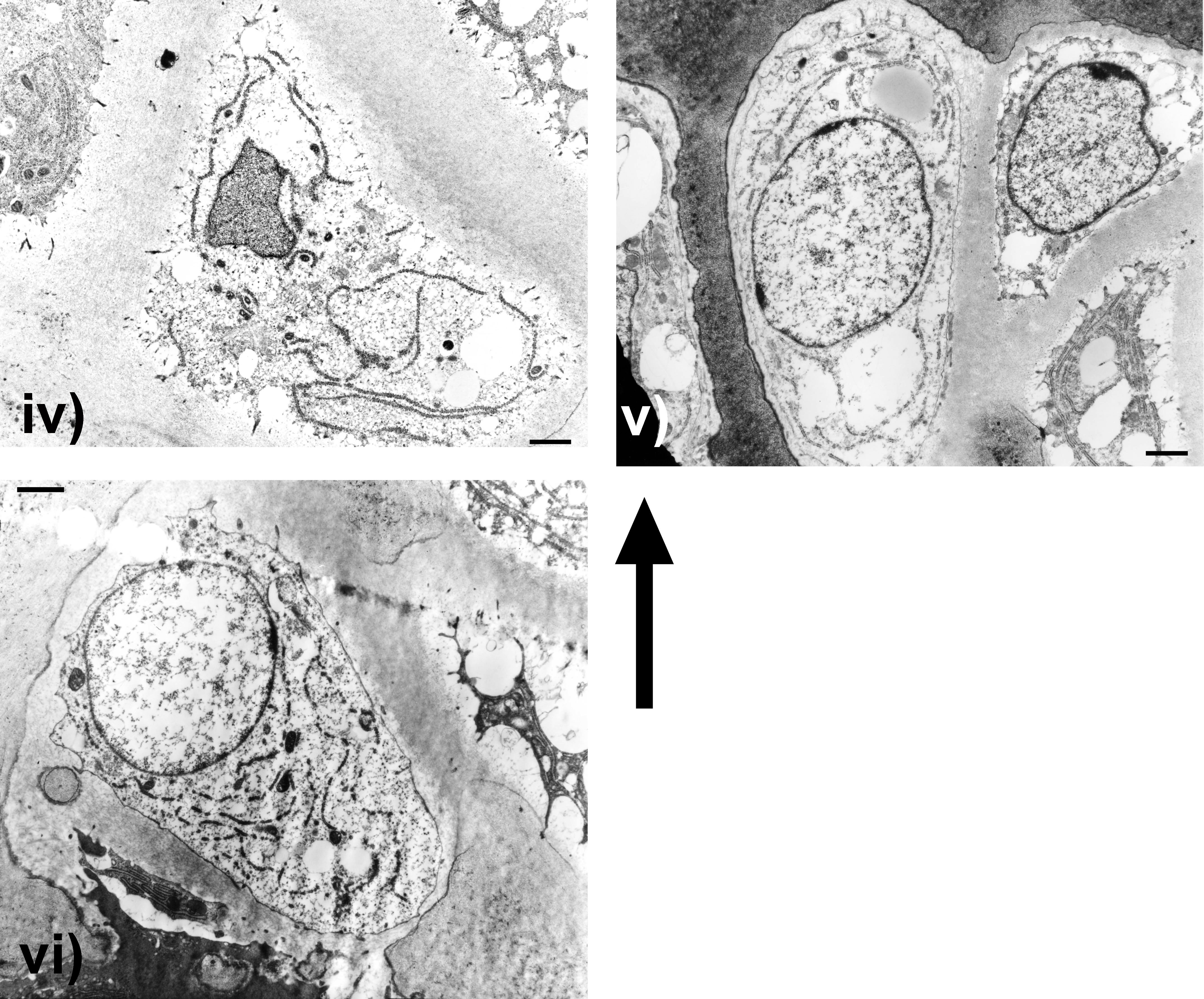

Supplement: Supplementary file 10 — Authors’ original file for figure 10 [file 12891_2014_2291_MOESM10_ESM.jpeg]

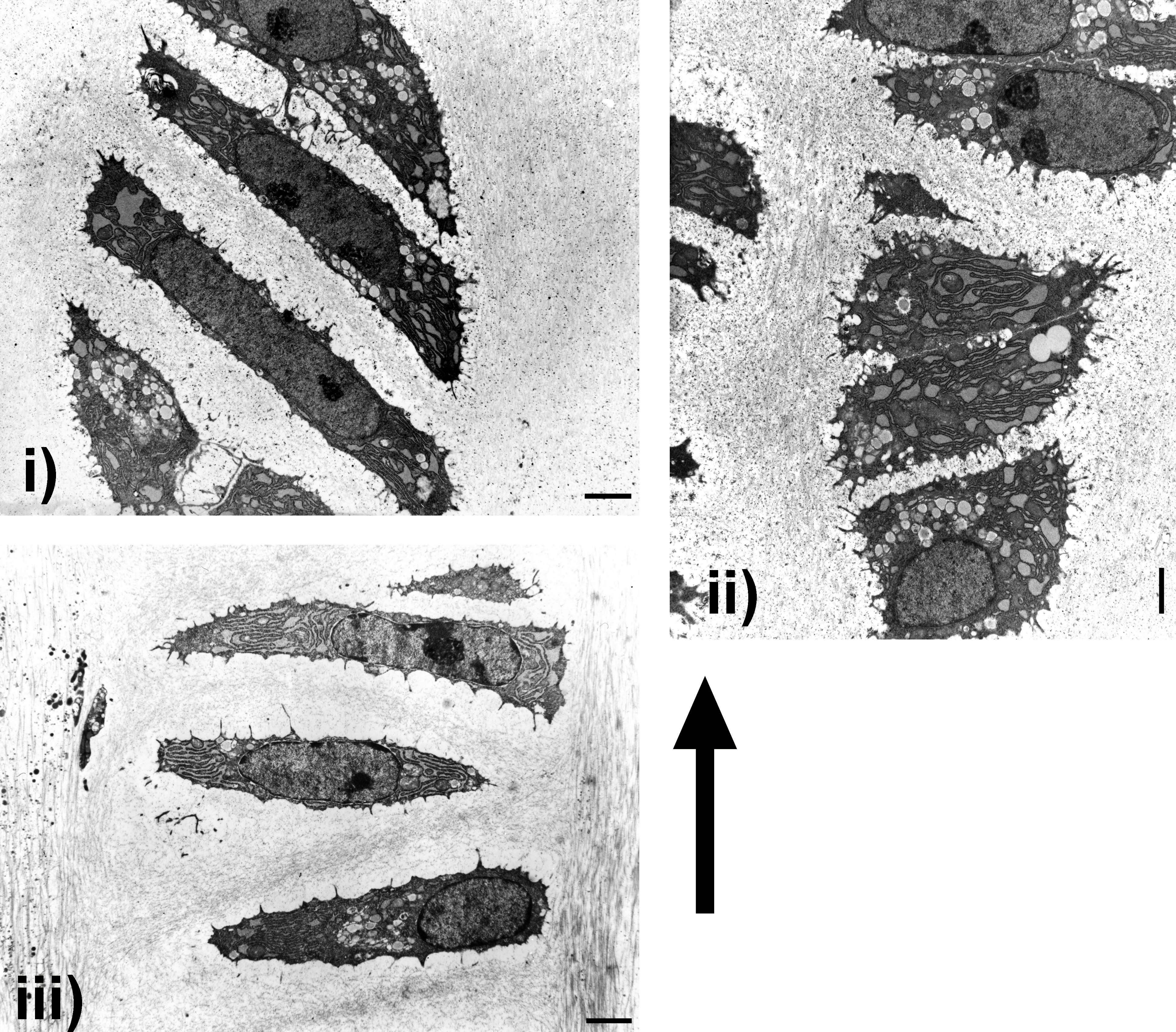

Supplement: Supplementary file 11 — Authors’ original file for figure 11 [file 12891_2014_2291_MOESM11_ESM.jpeg]

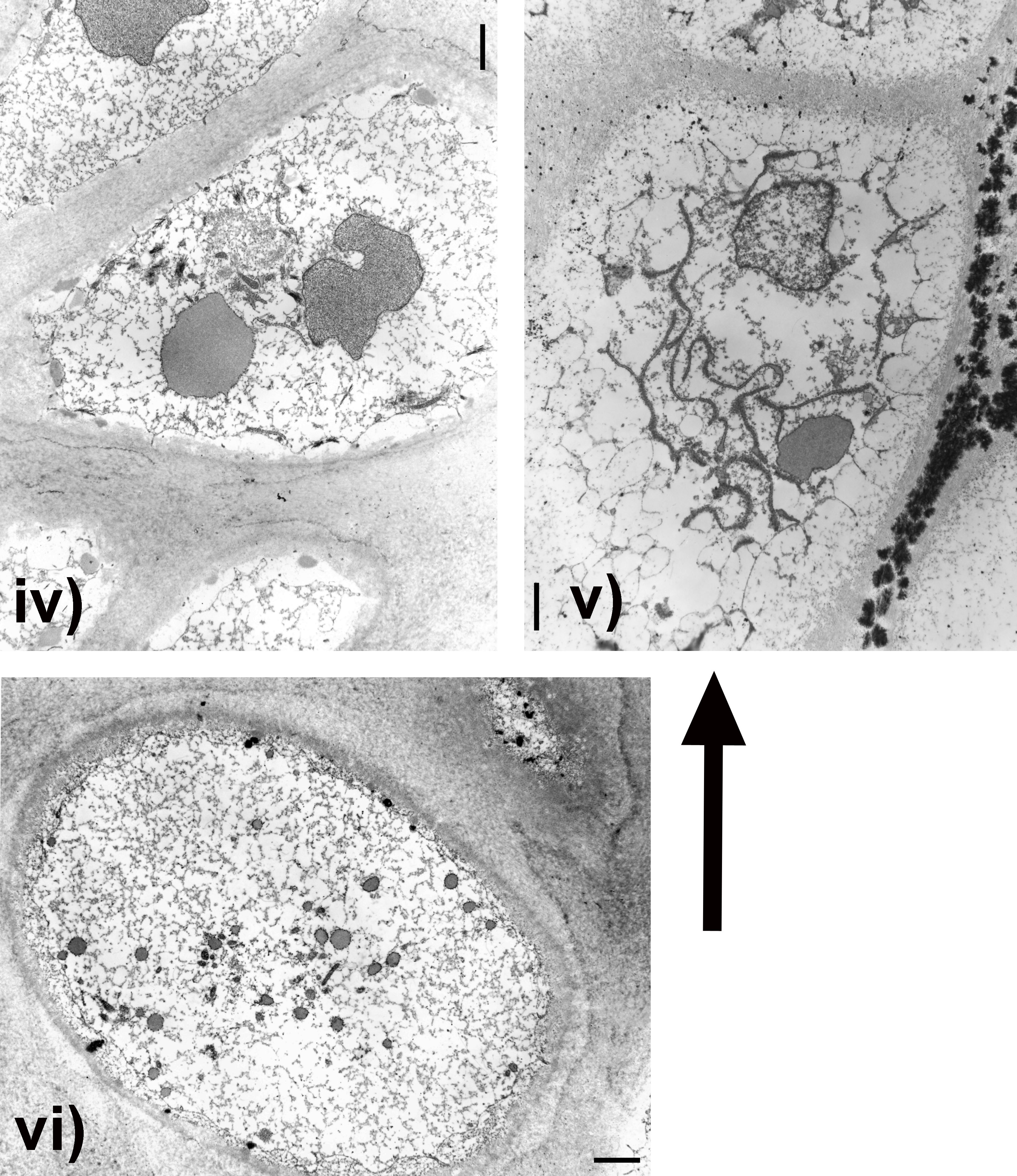

Supplement: Supplementary file 12 — Authors’ original file for figure 12 [file 12891_2014_2291_MOESM12_ESM.jpeg]
